# Differences in microbiota between acute and chronic perianal eczema

**DOI:** 10.1097/MD.0000000000025623

**Published:** 2021-04-23

**Authors:** Ming Ma, Hongmei Lu, Zuozhen Yang, Li Chen, Yingru Li, Xiu Zhang

**Affiliations:** a Department of Dermatology, Beijing Coloproctological Hospital, Beijing Erlonglu Hospital, Beijing; b MOE Laboratory of Biosystem Homeostasis and Protection, College of Life Sciences, Zhejiang University, Hangzhou, Zhejiang; c Cipher Gene LLC; d Department of Surgery; e Department of Internal Medicine, Beijing Coloproctological Hospital, Beijing Erlonglu Hospital, Beijing, China.

**Keywords:** 16S, microbiota, perianal eczema

## Abstract

Microbiota has been suggested to play a role in patients with intestinal and cutaneous diseases. However, the profiling of perianal eczema microbiota has not been described. We have explored the general profile and possible differences between acute and chronic perianal eczema. A total of 101 acute perianal eczema (APE) and 156 chronic perianal eczema (CPE) patients were enrolled in this study and the perianal microbiota was profiled via Illumina sequencing of the 16S rRNA V4 region.

The microbial α-diversity and structure are similar in APE and CPE patients; however, the perianal microbiota of the APE patients had a higher content of Staphylococcus (22.2%, *P* < .01) than that of CPE patients. Top10 genera accounting for more than 60% (68.81% for APE and 65.47% for CPE) of the whole microbiota, including *Prevotella*, *Streptococcus*, and *Bifidobacterium*, show an upregulation trend in the case of APE without reaching statistically significant differences. This study compared the microbiota profiles of acute and chronic perianal eczema. Our results suggest that the microbiota of acute perianal eczema patients is enriched in Staphylococcus compared with that in the chronic group. Our findings provide data for further studies.

## 1 Introduction

Perianal eczema is an allergic skin disease that occurs on the perianal skin and mucous membranes^[1]^ and can extend to the perineum and external genitalia. The risk factors of perianal eczema are complex. According to the previous studies, one of the causes of perianal eczema and pruritus ani may include the manifestations of nickel-induced systemic dermatitis.^[2]^ Patch test has shown that certain components in wet wipes, such as methylchloroisothiazolinone, are the main allergens of perianal eczema.^[3]^ The majority of typical clinical manifestations include severe itching and other symptoms, such as erythema, pimples, blisters, erosions, exudates, crusts caused by scratching, thickening, and roughening of the anus skin. It is difficult to treat perianal eczema because of the long and recurrent course of the disease,^[1]^ which has a negative effect on the physical and mental health of the patients. Clinical treatments are selected depending on different causes and skin lesions. Patients are supposed to avoid the contacts with the irritants and potential allergens. Low-potency corticosteroids or calcineurin inhibitors can be used for short-term local perianal treatment.^[4]^ It is safe and effective to treat perianal eczema with 0.1% topical tacrolimus.^[5]^ Long-term use of topical glucocorticoids inhibits the differentiation and proliferation of epidermal cells and weakens skin barrier and function.^[6]^ At present, there is no effective cure for perianal eczema. Previous studies have shown that the infection and colonization of skin microorganisms, especially *Staphylococcus aureus* (*S aureus)*, in the affected areas are the main reasons for the immune system disorder in patients with dermatitis and eczema^[7]^; there is a positive relationship between the number of *S aureus* colonies and the severity of skin lesion.^[8,9]^*S aureus* strains secrete enterotoxin and stimulate basophils to produce histamine and leukotriene.^[10]^ The normal microbiota of the intestinal tract is closely associated with human health. Imbalanced microbiota can cause a series of diseases, such as certain digestive system diseases, metabolic diseases, inflammatory bowel disease,^[11,12]^ irritable bowel syndrome,^[13,14]^ and autoimmune diseases.^[15–17]^ The anus is the gateway of the digestive tract to the external body, and perianal diseases are closely related to microorganisms. A study of bacteria cultured from the perianal skin swabs in patients with hematological diseases detected carbapenem-resistant Enterobacter (CRE), which provides an early sign of CRE bloodstream infections and antibacterial drug options for treatment of the disease.^[18]^ Therefore, detection of microorganism colonization in patients with perianal eczema and selecting sensitive antibiotics against possible pathogenic bacteria is of great significance for clinical treatment and prognostic evaluation.

## 2 Materials and methods

### 2.1 Study design

Patients newly diagnosed (from August 2018 to August 2019) with perianal eczema by a dermatologist were enrolled in the study. Inclusion criteria: diagnosis of perianal eczema, consent to join the microbiota research project, and understanding of the research purpose. Exclusion criteria: diagnosis with other digestive system diseases (such as colorectal cancer and diarrhea), serious cardiovascular diseases, pregnancy, preparation for pregnancy or lactation, infectious diseases, local skin trauma or infection, and inability to provide an informed consent. The study was approved by Beijing Coloproctological Hospital, and the participants provided written informed consent prior to participation.

### 2.2 Sample collection

Skin samples were collected from the perianal area (a single sample covering the whole space of the perianus) using sterile cotton swabs with sterile water. Each sample was rubbed 20 times with a cotton stick: 10 times in one direction and 10 times in a perpendicular direction. Microbiota sampling was conducted by the same group of investigators responsible for all study visits.

### 2.3 Genomics DNA extraction

Microbial genomic DNA was extracted by using a MagPure stool DNA KF kit B (Magen, China) according to the manufacturer's instructions. DNA was quantified with a Qubit fluorometer by using a Qubit dsDNA BR assay kit (Invitrogen), and the quality of DNA was checked by 1% agarose gel electrophoresis.

### 2.4 Library construction

Bacterial V4 regions of the 16S rRNA gene were amplified with primers 515F (5’-GTGCCAGCMGCCGCGGTAA-3’) and 806R (5’-GGACTACHVGGGTWTCTAAT-3’). Forward and reverse primers were tagged with the Illumina adapter, pad and linker sequences. PCR was performed in a 50 μL reaction containing 30 ng template, fusion PCR primers and PCR master mix.

PCR cycling conditions were as follows: 95°C for 3 minutes, 30 cycles at 95°C for 45 seconds, 56°C for 45 seconds, and 72°C for 45 seconds, and a final extension for 10 minutes at 72°C. The PCR products were purified using Agencourt AMPure XP beads and eluted using an elution buffer. Libraries were qualified by an Agilent Technologies 2100 bioanalyzer. The validated libraries were used for sequencing on an Illumina HiSeq 2500 platform following the standard Illumina pipelines to generate 2 × 250 bp paired-end reads.

### 2.5 Sequencing and bioinformatics analysis

Raw reads were filtered to remove the adaptors and low-quality and ambiguous bases, and the paired-end reads were added to the tags by the Fast Length Adjustment of Short reads software (FLASH, v1.2.11)^[19]^ to obtain the tags. The tags were clustered into operational taxonomic unit (OTU) with a cutoff value of 97% using UPARSE software (v7.0.1090)^[20]^ and chimera sequences were compared with the Gold database using UCHIME (v4.2.40).^[21]^ Then, the representative OTU sequences were taxonomically classified using Ribosomal Database Project (RDP) Classifier v.2.2 with a minimum confidence threshold of 0.6 and trained on the Greengenes database v201305 by QIIME v1.8.0.^[22]^ The USEARCH_global^[23]^ was used to compare all the tags with original OTU to obtain an OTU abundance statistics table of each sample.

Alpha and beta diversity were estimated by MOTHUR (v1.31.2)^[24]^ and QIIME (v1.8.0)^[22]^ at the OTU level. Sample clustering was performed by QIIME (v1.8.0)^[22]^ based on UPGMA. The KEGG and COG functions were predicted using the PICRUSt software.^[25]^ Bar plots and heatmaps of various classification levels were plotted with R package v3.4.1 and R package “gplots,” respectively.

Phylogenetic tree of the species was constructed using FastTree (v2.1.3)^[26]^; Principal Coordinate Analysis (PCoA) was performed by QIIME (v1.8.0)^[22]^; UPGMA cluster and abundance mapping was performed by phytools and R package version 3.5.1. LEfSe cluster or LDA analysis were conducted by LEfSe. Significant Species or function were determined by R (v3.4.1) based on the Wilcoxon test or the Kruskal test. For all statistical analyses, two-sided *P* < .05 was considered statistically significant.

### 2.6 Statement of ethics

The study was approved by Beijing Coloproctological Hospital, and the participants provided written informed consent prior to participation.

## 3 Results

### 3.1 Subjects

**Figure 1:**
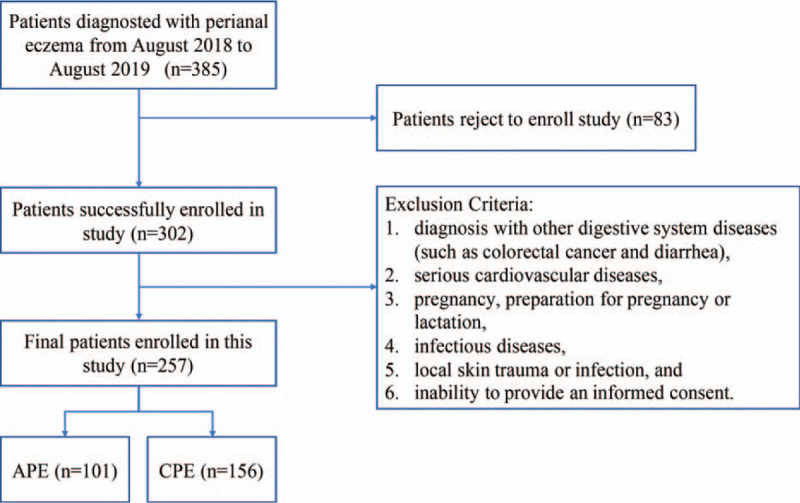
Flowchart for inclusion and exclusion criteria.

A total of 257 subjects, including 101 APE and 156 CPE patients, were recruited in this study; the flow chart of study design was draw with exclusion criterion (Fig. 1), demographic characteristics are presented in Table 1 (see Fig., Supplemental Digital Content Fig. S1, Table 1). There were no significant differences in gender and age between the APE and CPE groups.

**Table 1:** Characteristics of the study participants.

	APE (n = 101)	CPE (n = 156)	*P*
Gender (male, %)	48 (47.5%)	73 (46.8%)	.9093
Age	41.07 ± 12.06	43.06 ± 12.08	.1985

### 3.2 Microbial α-diversity in the APE and CPE groups

We obtained a total of 19,248,569 high-quality tags by quality filtering with the coverage over 99.0%. The obtained sequences per sample were clustered to 2213 OTUs. The results of bacterial community richness and diversity are shown in Table 2. There was no difference in sobs, coverage, Shannon, Simpson, ace, or chao indexes between the 2 groups (see Figure, Supplemental Digital Content Fig. S2).

**Table 2:** The index of Alpha diversity.

	APE (n = 101)	CPE (n = 156)	*P*
Sobs	291.78218 ± 135.16002	292.05769 ± 144.86328	.67944
Chao	356.07109 ± 153.14152	357.66587 ± 168.49536	.80858
Ace	362.40699 ± 151.18454	363.62429 ± 168.60646	.73177
Shannon	3.20243 ± 0.72045	3.25394 ± 0.74833	.44402
Simpson	0.11752 ± 0.09936	0.10866 ± 0.09326	.20266
Coverage	0.99894 ± 0.00048	0.99892 ± 0.00061	.89271

The similarity of the bacterial community structures between the APE and CPE groups was evaluated by PCoA (see Fig., Supplemental Digital Content Fig. S3). There were no detectable differences in the microbiota structure between the 2 groups. ANOSIM was performed and detected a high similarity in the bacterial communities between the APE and CPE patients based on 2 algorithms (unweighted unifrac, *r* = −0.012, *P* = .7439; and weighted unifrac *r* = 0.013, *P* = .1772, respectively). The theta YC was also evaluated, there were no significant difference between groups (*R* = 0.0003, *P* = .368, see Fig., Supplemental Digital Content Fig. S4).

### 3.3 Taxa abundance in microbiota of APE and CPE patients

**Figure 2:**
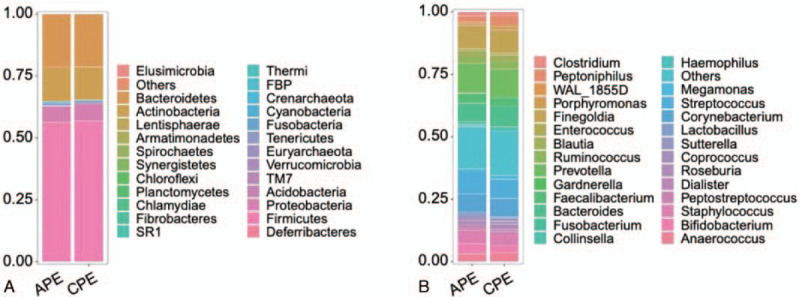
Relative abundance in APE and CPE. A. Phylum; B. Genus. APE = acute perianal eczema, CPE = chronic perianal eczema.

**Figure 3:**
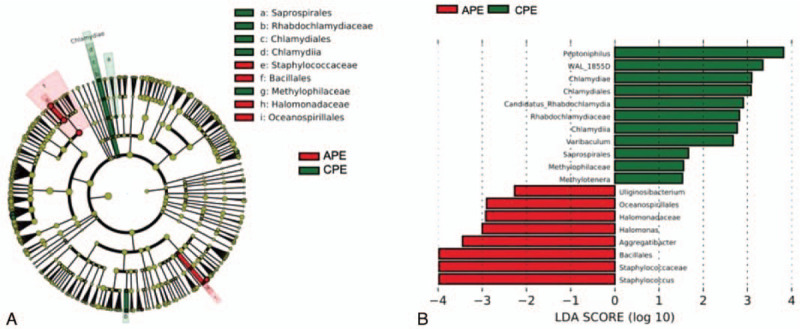
Microbes with the highest differential abundance in APE vs CPE based on LEfSe analysis. Red box: APE; green box: CPE. A. Cluster map of differentially expressed microbes according to LEFSe. B. Latent Dirichlet Allocation (LDA) map for significantly different microbes according to LEFSe. APE = acute perianal eczema, CPE = chronic perianal eczema.

**Figure 4:**
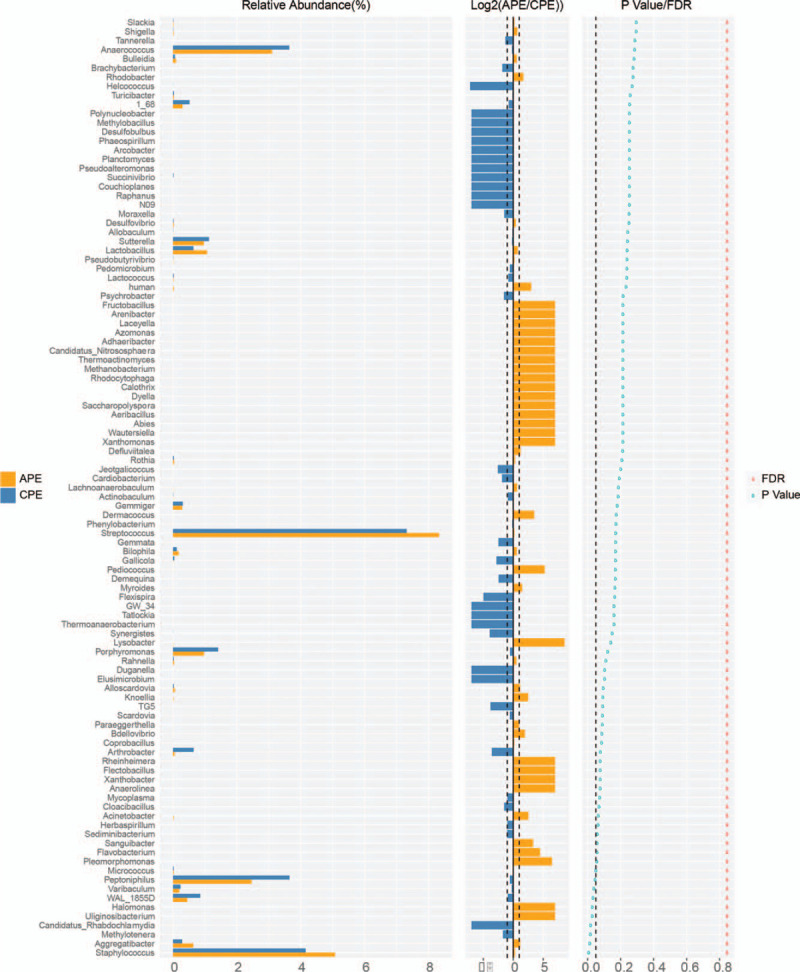
Differentially expressed genera between APE and CPE according to Wilcoxon rank-sum test. Species abundance is shown on the left. Average expression ratio for APE/CPE (log2 transformed) is shown in the middle; cutoff line is two-fold (upregulated or downregulated). *P* values and FDR are shown on the right; cutoff for *P* value is .05. APE = acute perianal eczema, CPE = chronic perianal eczema.

The majority of the perianal bacteria detected in this study falls into 3 phyla: Firmicutes, Bacteroidetes, and Actinobacteria (Fig. 2a). The main genera of the perianal microbiota (the percentage over 1%) include 19 genera that comprise up to 76.6% of the total microbiota, such as *Bacteroides*, *Prevotella*, *Finegoldia*, *Streptococcus*, *Corynebacterium*, *Staphylococcus*, *Bifidobacterium*, and *Faecalibacterium* (Fig. 2b). At the genus level, *Staphylococcus*, *Staphylococcaceae*, *Bacillales*, *Aggregatibacter*, *Halomonas*, *Halomonadaceae*, and *Oceanspirillales* were significantly enriched in the APE group, while *Peptoniphilus*, *WAL_1855D*, *Chlamydiae*, *Candidatus, Rhabdchlamydia*, and *Rhabdochlamydiaceae* had relatively higher abundance in the CPE group (Fig. 3). The taxa that have the highest likelihood to be different between the APE and CPE groups were identified by LEfSe (Fig. 3). Wilcoxon rank-sum test was performed to explore the differences between the APE and CEP groups at the overall species level, relative abundance, and ratio of APE/CPE (Fig. 4).

**Figure 5:**
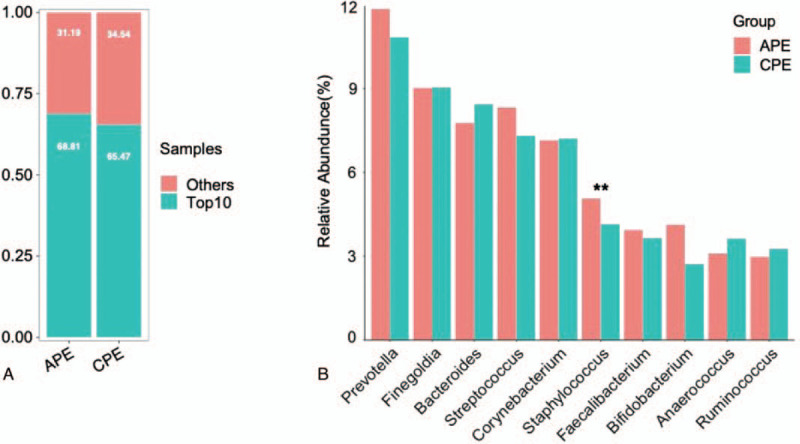
Top10 genera abundance in APE vs CPE. A. Top10 genera abundance in total microbiota. B. Top10 genera abundance between APE and CPE. ^∗∗^: *P* < .01. APE = acute perianal eczema, CPE = chronic perianal eczema.

Top10 genera accounting for more than 60% (68.81% for APE and 65.47% for CPE) of the whole microbiota (Fig. 5), *Prevotella*, *Streptococcus*, and *Bifidobacterium*, have an upregulation trend in the APE group; however, all *P* values were higher than .05. Comprehensive consideration of significant changes and abundance in the total microbiota (the percentage over 1% and inclusion into top10 genera) finally identified that *Staphylococcus* was significantly changed between the APE and CPE patients (upregulation by 22.2%, *P* < .01).

## 4 Discussion

In this study, we uncovered the profiles of the microbial communities around the perianal area in patients with acute perianal eczema and chronic perianal eczema. Additionally, we found that the abundance of *Staphylococcus* was significantly different between the APE and CPE groups. Previous studies showed that microbiota plays important roles in skin diseases, such as rosacea^[27,28]^ and acne,^[29]^ and is associated with disease severity in pediatric atopic dermatitis,^[30,31]^ irritant contact dermatitis and allergic contact dermatitis.^[32]^ Importantly, low diversity of gut microbiota in the infants was found in atopic eczema^[33]^ thus implying that the abundance of each microbe and diversity of the whole microbiota contribute to skin diseases.

The anus is the gateway of the digestive tract to the external body; eczema around the perianal area may be influenced by gut microbes. The number of studies profiling perianal microbiota is limited and the information on microbial abundance and diversity is not available. It is known that acute perianal eczema is characterized by erythema, pimples, blisters, exudates and crusts caused by scratching; typical manifestation of chronic perianal eczema includes skin lesions, such as thickening, roughness and the presence of scales. Theoretically, the microbiota should differ between acute and chronic perianal eczema; however, the details of the differences between these conditions are not known. Assessment of microbial abundance and identification of the species may enable personalized treatment of the patients similar to precision medicine in cancer.

Our data have detected that *Staphylococcus* is upregulated in acute perianal eczema. Previous studies on *Staphylococcus* mainly focused on the microevolution and epidemiology in atopic eczema,^[34]^ severity of hand eczema,^[35]^ enterotoxin induction of histamine and leukotriene release,^[10]^ and intervention to reduce *Staphylococcus* to manage eczema.^[36,37]^ Li group investigation focused on *Staphylococcus* in hand eczema and determined that *S aureus* colonization plays important roles in morbidity and progression of chronic hand eczema, which correlated with chronicity and severity of the disease.^[38]^ In our study, the abundance of *Staphylococcus* in acute perianal eczema is higher than that in chronic perianal eczema. We also detected the upregulation trends of *Prevotella*, *Streptococcus*, and *Bifidobacterium* in acute perianal eczema. However, the trends were not statistically significant.

## Acknowledgments

We thank Cipher Gene LCC for assistance with sequencing.

## Author contributions

**Conceptualization:** Xiu Zhang.

**Data curation:** Ming Ma, Hongmei Lu.

**Formal analysis:** Zuozhen Yang.

**Funding acquisition:** Xiu Zhang.

**Investigation:** Ming Ma, Hongmei Lu, Xiu Zhang.

**Software:** Zuozhen Yang.

**Writing – original draft:** Ming Ma, Hongmei Lu.

**Writing – review & editing:** Li Chen, Yingru Li.

## Supplementary Material

Supplemental Digital Content

## Supplementary Material

Supplemental Digital Content

## Supplementary Material

Supplemental Digital Content

## Supplementary Material

Supplemental Digital Content
