# Supplementary material for: Differences in microbiota between acute and chronic perianal eczema
Source: Medicine (Baltimore). 2021 Apr 23;100(16):e25623. doi: 10.1097/MD.0000000000025623 (PMC8078401; doi:10.1097/MD.0000000000025623)
Supplement: Supplemental Digital Content [file medi-100-e25623-s001.doc]

Figure S1 Characteristics of the enrolled study participants

Age distribution is shown on the left. Gender information is shown in the middle. The number of patients in APE/CPE group is on the right. Red color: APE (acute perianal eczema); green color: CPE (chronic perianal eczema)
