# Supplementary material for: Differences in microbiota between acute and chronic perianal eczema
Source: Medicine (Baltimore). 2021 Apr 23;100(16):e25623. doi: 10.1097/MD.0000000000025623 (PMC8078401; doi:10.1097/MD.0000000000025623)
Supplement: Supplemental Digital Content [file medi-100-e25623-s002.doc]

Figure S2 Alpha diversity distribution between APE and CPE

Alpha diversity distribution between APE and CPE. Red box: APE, blue box: CPE. APE: acute perianal eczema; CPE: chronic perianal eczema
