# Supplementary material for: Differences in microbiota between acute and chronic perianal eczema
Source: Medicine (Baltimore). 2021 Apr 23;100(16):e25623. doi: 10.1097/MD.0000000000025623 (PMC8078401; doi:10.1097/MD.0000000000025623)
Supplement: Supplemental Digital Content [file medi-100-e25623-s003.doc]

Figure S3 PCoA analysis of the microbiota in the APE and CPE groups. Red ball: APE, blue ball: CPE. a: unweighted unifrac PCoA, b: weighted unifrac PCoA. APE: acute perianal eczema; CPE: chronic perianal eczema.
